# Understanding what matters most to patients in acute care in seven countries, using the flash mob study design

**DOI:** 10.1186/s12913-021-06459-4

**Published:** 2021-05-19

**Authors:** Eva S. van den Ende, Bo Schouten, Marjolein N. T. Kremers, Tim Cooksley, Chris P. Subbe, Immo Weichert, Louise S. van Galen, Harm R. Haak, John Kellett, Jelmer Alsma, Victoria Siegrist, Mark Holland, Erika F. Christensen, Colin A. Graham, L. E. U. N. G. Ling Yan, Line E. Laugesen, Hanneke Merten, Fraz Mir, Rachel M. Kidney, Mikkel Brabrand, Prabath W. B. Nanayakkara, Christian H. Nickel, on behalf of all local collaborators, Vibe Maria Laden Nielsen, Karen Vestergaard Andersen, Hanne Nygaard, Kasper Karmark Iversen, Martin Schultz, Peter Hallas, Magnus Peter Brammer Kreiberg, Line Emilie Laugesen, Anne Mette Green, Tanja Mose Kristensen, Helene Skjøt-Arkil, Hejdi Gamst-Jensen, Torbjørn Shields Thomsen, Camilla Dahl Nielsen, Kristian Møller Jensen, Søren Nygaard Hansen, Marc Ludwig, Henriette Sloth Høg, Dorthe Gaby Bove, Vibe Kristine Sommer Mikkelsen, Sune Laugesen, Nerma Todorovac, Stine Nørris Nielsen, Poul Petersen, Hanna Karstensen, Gitte Boier Tygesen, Rasmus Aabling, Lone Pedersen, Sef J. L. W. Van Den Beuken, Ditte Høgsgaard, Thomas Christophersen, Christina Smedegaard, Mette Worsøe, Marie-Laure M. A. Bouchy Jacobsson, Le Elias Lyngholm, Sara Fonager Lindholm, J. M. van Pelt-Sprangers, Ralph K. L. So, Sander Anten, Judith van den Besselaar, Gerba Buunk, Lorenzo Romano, Daan Eeftick Schattenkerk, Frits Holleman, Rishi S. Nannan Panday, Sacha C. Rowling, Michiel Schinkel, Sophie van Benthum, S. J. J. Logtenberg, Esther M. G. Jacobs, Jelmer Alsma, William Boogers, Marlies Verhoeff, Barbara V. van Munster, Emma Gans, Noortje Briët-Schipper, Yotam Raz, Ayesha Lavell, Fatima El Morabit, Gert-Jan Timmers, Ad Dees, Ginette Carels, Berit Snijer, Anne Floor Heitz, Pim A. J. Keurlings, Susan Deenen, Patricia M. Stassen, Hajar Kabboue, Ineke Schouten, C. E. H. Siegert, Jacobien J. Hoogerwerf, Lianne de Kleijn, Frank H. Bosch, Annebel Govers, Bianca van den Corput, H. S. Noordzij-Nooteboom, M. J. Dekkers, Annemarie van den Berg, Jan C. ter Maaten, Dennis G. Barten, Tessel Zaalberg, John Soong, Norshima Nashi, Louise S. van Galen, Lim Wan Tin, Tharmmambal Balakrishnan, Siti Khadijah Binte Zainuddin, Christian H. Nickel, Victoria Siegrist, Fraz Mir, Channa Vasanth Nadarajah, Aled Lewis, David Ward, C. Weerasekera, Thandar Soe, Thomas Cozens, Joanne McDonald, Mark Holland, Andrew Down, Immo Weichert, Harith Altemimi, Tim Cooksley, A. Seccombe, Chris P. Subbe, Ben Lovell, Colin Graham, Ronson Lo, Ling Leung, Rachel M. Kidney

**Affiliations:** 1 Section of Acute Medicine, Department of Internal Medicine, Amsterdam Public Health research institute, Amsterdam University Medical Center, location VU University Medical Center, De Boelelaan 1117, 1081 HV Amsterdam, ZH The Netherlands; 2 Department of Public and Occupational Health, Amsterdam Public Health research institute, Amsterdam University Medical Center, location VU University Medical Center, Amsterdam, the Netherlands; 3 Department of Health Services Research, CAPHRI School for Public Health and Primary Care, Aging and Long Term Care, Maastricht University, Maastricht, the Netherlands; 4 Department of Internal Medicine, Máxima MC, Veldhoven/Eindhoven, The Netherlands; 5 Department of Acute Medicine, University Hospital of South Manchester, Manchester, UK; 6 Department of Acute Medicine, Ysbyty Gwynedd Hospital, Bangor, UK; 7 School of Medical Sciences, Bangor University, Bangor, UK; 8 Department of Acute Medicine, Ipswich Hospital, East Suffolk and North Essex NHS Foundation Trust Ipswich Hospital, Ipswich, UK; 9 Department of Internal Medicine, Division of General Internal Medicine, Maastricht University Medical Center, Maastricht, The Netherlands; 10 Department of Emergency Medicine, Hospital of South West Jutland, Esbjerg, Denmark; 11 Department of Internal Medicine, Erasmus University Medical Center, Rotterdam, the Netherlands; 12 Department of Cognitive and Decision Sciences, University of Basel, Basel, Switzerland; 13 Department of Emergency Medicine, University Hospital Basel, Basel, Switzerland; 14 Section of Acute Medicine, Department of Internal Medicine, Salford Royal NHS Foundation Trust, Salford, UK; 15 Center for Prehospital and Emergency Research, Clinic of Internal and Emergency Medicine, Aalborg University Hospital and Aalborg University, Aalborg, Denmark; 16 Institute of Clinical Medicine, Aalborg University Hospital and Aalborg University, Aalborg, Denmark; 17 Department of Emergency Medicine, Chinese University of Hong Kong, Hong Kong, Hong Kong; 18 Department of Medicine, Addenbrooke’s Hospital, Cambridge, UK; 19 Department of Internal Medicine, St. James’s Hospital, Dublin, Ireland; 20 Department of Emergency Medicine, Odense University Hospital, Odense, Denmark

**Keywords:** What matters most, Patient-centred care, Acute care, Emergency medicine, Quality of care, Patient-physician communication, Research methods

## Abstract

**Background:**

Truly patient-centred care needs to be aligned with what patients consider important, and is highly desirable in the first 24 h of an acute admission, as many decisions are made during this period. However, there is limited knowledge on what matters most to patients in this phase of their hospital stay. The objective of this study was to identify what mattered most to patients in acute care and to assess the patient perspective as to whether their treating doctors were aware of this.

**Methods:**

This was a large-scale, qualitative, flash mob study, conducted simultaneously in sixty-six hospitals in seven countries, starting November 14th 2018, ending 50 h later. One thousand eight hundred fifty adults in the first 24 h of an acute medical admission were interviewed on what mattered most to them, why this mattered and whether they felt the treating doctor was aware of this.

**Results:**

The most reported answers to “*what* matters most (and why)?” were ‘*getting better or being in good health’* (why: to be with family/friends or pick-up life again)*, ‘getting home’* (why: more comfortable at home or to take care of someone) and *‘having a diagnosis’* (why: to feel less anxious or insecure)*.* Of all patients, 51.9% felt the treating doctor did not know what mattered most to them.

**Conclusions:**

The priorities for acutely admitted patients were ostensibly disease- and care-oriented and thus in line with the hospitals’ own priorities. However, answers to why these were important were diverse, more personal, and often related to psychological well-being and relations. A large group of patients felt their treating doctor did not know what mattered most to them. Explicitly asking patients what is important and why, could help healthcare professionals to get to know the person behind the patient, which is essential in delivering patient-centred care.

**Trial registration:**

NTR (Netherlands Trial Register) NTR7538.

**Supplementary Information:**

The online version contains supplementary material available at 10.1186/s12913-021-06459-4.

## Key points

- To deliver patient-centred care, it is important to know what matters to every patient. Nevertheless, our study showed that a large group of patients felt that their treating physician did not know what mattered most to them at that moment.
- Although the majority of patients initially indicated disease- and care-related matters to be most important, they shared diverse personal stories when asked about their motivations and why these were important. These stories show the person behind the patient.
- The questions “What matters most to you?” and especially “why does this matters most?” are questions that can provide healthcare workers with personal information about the patients’ preferences, needs, goals, values and emotions, necessary to deliver patient-centred care.

## Introduction

Effective patient-doctor communication and patient involvement can lead to increased patient satisfaction, better health outcomes, and is essential to the delivery of patient-centred care [1, 2]. However, with growing worldwide pressure on acute healthcare systems and the resultant limited time available per patient [3, 4], it is increasingly challenging for healthcare providers to have comprehensive conversations with patients. As a result, they may not have adequate psychological and emotional insights into the patients’ priorities [5, 6]. Research shows that many clinicians’ conversations are *about* patients and not *with* them [7], and that patients are seen as their disease(s) rather than as individuals [6].

The goal of patient-centred care is to customize care to the individual patient, taking into consideration their preferences, needs and values. To achieve this, Barry and Edgman-Levitan (2012) proposed asking the patient “what matters to you?”, in addition to “what is the matter?” [8]. This topic has received increasing attention over the years, and an annual international “What Matters to you?” day was launched in 2016 to promote meaningful conversations between healthcare providers and patients [9]. The Institute for Healthcare Improvement (IHI) states that the “what matters to you?” question is a quick, simple, but yet profound way to start deep and personal conversations with patients [10]. It encompasses discussing the patients’ priorities and values alongside potentially revealing unanswered questions, which could provide input for a personalized care plan [11].

Much research has been conducted to investigate the priorities and preferences of patients with specific diagnoses [12–14], treated in the Emergency Department [15] or in chronic disease programs [16–20], which has resulted in the development of multiple frameworks (e.g. Lim [21] and Picker experience [22]). However, little is known about what is most important to the heterogeneous group of patients (with regards to morbidity, basic characteristics, culture, health and socio-economic status) during the acute phase of a hospital admission. The first 24 h of an acute admission will often determine the course of the hospital stay. In this phase many diagnostic tests are carried out, care plans are created, and key decisions made. It is crucial that during this time-period the priorities of the patient are clear to the healthcare team [12]. Therefore, the primary objective of this study was to identify and categorize what matters most to the diverse group of patients in the first 24 h of an admission.

Not only must doctors converse with patients, it is important that patients feel that they have been listened to, have been understood, and that their concerns will be considered and addressed [5, 8, 23]. As such, the secondary objective of this study was to assess the patient perspective on whether they felt their doctor knew what mattered most to them.

## Methods

### Study design and setting

A large-scale qualitative international study was conducted using the flash mob research design [24, 25]. The flash mob research design is based on the concept of flash mobs, where groups of people suddenly meet in a public place, briefly perform a specific act and then quickly disappear. This allowed us to collect structured qualitative data from a large number of patients within a short time-period. To get an overview of what matters most to patients in a wider socio-cultural context, the study was conducted across a wide range of countries, regions and cultures.

The study started on November 14th, 2018 at 10 AM local time, and ended 50 h later on November 16th, 12 PM local time. Patients in 66 hospitals were recruited simultaneously in The Netherlands, United Kingdom, Ireland, Denmark, Switzerland, Hong Kong and Singapore. Data were collected in acute medical units (AMUs, short stay departments [26]) and other medical wards (i.e. observation units, cardiology, geriatrics, gastroenterology, haematology, internal medicine, nephrology, neurology, oncology, pulmonary medicine and rheumatology).

The Executive Committee of the Medical Ethics Review Committee of VU University Medical Center (IRB00002991) reviewed the research proposal, approved the project and decided that the Medical Research involving Human Subjects Act did not apply (reference No. 2018.318). In all other countries, approval of national ethics committees and executive boards was sought in line with local research policies.

The acute medicine research team of Amsterdam University Medical Center (located at VUmc, the Netherlands) coordinated the project. Collaborators from the Safer@Home research consortium were involved in the design of the study and acted as coordinating researchers, responsible for the recruitment of hospitals in their country [27].

### Research team and responsibilities

The coordinating investigator in each country was responsible for translating the English datasheet into the local language (using forward- and backward translation, according to the ISPOR guidelines [28]) and translating the open text answers to English (with a forward- and backward translation of a 10% convenience sample).

Every hospital had one ambassador responsible for appointing interviewers for data collection, recruitment of patients and entering the data into the digitalized secured database (Castor EDC). Interviewers were physicians, (research) nurses, medical students, or psychologists, all trained in communication skills.

### Recruitment of patients

Consecutive sampling was used to recruit a broad range of participants which would be largely representative of the acute patient population. All patients were 18 years or older, were unplanned admitted to hospital in the previous 24 h and able to give informed consent. Patients presenting with surgical, trauma and obstetric conditions and patients unable to give informed consent, as judged by the medical team, were excluded. Patients were asked for oral or written informed consent, depending on national research policies. Patients were approached face-to-face and assured that their decision to participate or not participate would have no consequences for their care.

### Questionnaire

In the questionnaire we used the classic ‘what matters to you?’ question [8, 10, 29, 30]. After a pilot study in ten patients, we found that adding a probing question (‘why is this important to you?’) was necessary to grasp the full concept. The data from these patients were used purely for the purpose of pilot testing the questionnaire, and not included in the data analysis.

The question ‘does your treating doctor in the hospital know what matters to you most?’ was added to find out about the patients’ perception regarding this subject. The questionnaire was complemented by questions concerning basic characteristics, living conditions, social and work situation. To find out how patients interpreted all questions, we used a cognitive interviewing style [31] during the pilot (e.g. by asking their opinion about the content and relevance of questions).

All questionnaires were available in each country’s local language.

### Data collection and privacy

Interviewers solely introduced themselves by name and had no prior relationship with the patients. Each interview took approximately 5 min. Data were collected at the bedside, and either entered directly into the digital database or transcribed from a paper datasheet, without the use of audio or visual recordings. Patients’ responses were not recorded verbatim, but paraphrased by the interviewer. Paraphrased answers were not returned to patients for review.

All interviewers had their own personal Castor EDC account for data input and were trained by both video tutorials and written instructions. Measures and warnings were built into the database to minimize the potential for errors. Interviewers transcribed the patient’s answers into the Castor EDC database. All records were labelled with an individual number. The key list with record numbers could only be accessed by the local coordinating researcher. No directly identifiable data were entered into the database.

### Data translation and development of the conceptual coding framework for content analysis

Danish, Swiss and Dutch data were translated to English; back-translation was conducted on 10% convenience samples and checked by independent assessors. No essential differences between the original data and back-translations were found.

To analyse the large number of open-text answers, a framework needed to be developed that could be used for coding both the answers to the ‘what matters most?’ and ‘why?’ questions.

An inductive approach of content analysis was used to identify categories and sub-categories in the data, leading to the development of a conceptual framework on what matters most to acutely admitted patients and why [32]. This framework was developed through five phases (using open coding, grouping, categorization and abstraction throughout each phase [32]), by four researchers (two medical doctors and two psychologists). A detailed description of the process can be found in Figure S1 in the Supplementary Material.

### Coding and data analysis

All 3700 answers (100% of data) to the ‘what matters most?’ and ‘why?’ questions were independently coded by both a medical doctor (EE or MK) and a psychologist (BS or HM), using the developed framework. Multiple categories could be assigned to one answer, without hierarchy. When there were discrepancies in assigned categories, an extensive consensus procedure followed (resulting in 100% agreement regarding the final categories). Composition of teams rotated to account for differences in interpretation (i.e. EE + BS, EE + HM, MK + BS, MK + HM).

As the qualitative data were large-scale, the frequency of categories was analysed and visualized in word clouds. Moreover, we analysed the combined occurrence of answers to the ‘what matters most to you?’ and ‘why?’ questions to identify patterns. We did this by counting which combinations of categories occurred most between the ‘what matters most?’ and ‘why?’ question (for example; patients often wanted to go home because they missed family members). Finally, we performed multiple subgroup analyses.

Coding was performed in Excel (Microsoft Office Professional Plus 2016). Word counts and word clouds were generated using Atlas.ti8 (Atlas.ti Scientific Software GmbH). Descriptive statistics were performed with SPSS for Windows, version 24 (SPSS Inc).

## Results

**Fig. 1:**
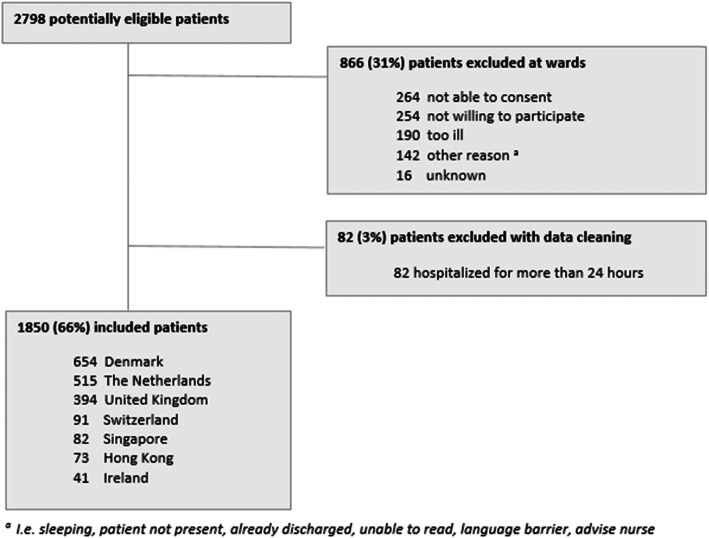
Patients included and excluded in analysis

During the inclusion period, 2798 patients had been admitted to the participating units for 24 h or less, and were therefore eligible for inclusion. However, 866 (31%) patients were excluded because they were not able to give informed consent or were unwilling or unable to participate (Fig. 1). Eighty-two patients were interviewed but later excluded because they had been admitted for more than 24 h prior to their questionnaire. Therefore, the interviews of 1850 (66%) acutely admitted patients were analysed. Figure 1 provides an overview of the inclusion process and numbers of included patients per country*.* Table 1 shows the patient characteristics of the included patients.

**Table 1:** Characteristics of 1850 included patients

Characteristics ^a^	No. (%) ^b^
**Sex (*n*** **= 1836, 14 missing)**	
Male	*918 (50.0)*
Female	*918 (50.0)*
**Age in 5 year intervals, median (IQR)**	*66–70 (51–55 - 76-80)*
**Patient had children (*n*** **= 1843, 7 missing)**	
Yes	*1466 (79.2)*
No	*366 (19.9)*
I prefer not to tell	*11 (0.6)*
**Patient had pets (*n*** **= 1838, 12 missing)**	
Yes	*559 (30.4)*
No	*1279 (69.6)*
**Work situation (*n*** **= 1850, 0 missing)** ^c^	
Retired	*1083 (58.5)*
Employed by a company	*378 (20.4)*
Unemployed but not retired	*253 (13.7)*
Self employed	*100 (5.4)*
Studying	*36 (0.2)*
**Living condition (*n*** **= 1840, 10 missing)** ^c^	
With partner or family	*1181 (64.2)*
Alone	578 (31.4)
Healthcare facility, of which	81 (4.4)
Retirement home	43 (53.1)
Nursing home	18 (22.2)
Rehabilitation centre	2 (2.5)
Other	18 (22.2)
**Help at home (*n*** **= 1761, 89 missing)**	
No	1248 (70.9)
Yes, of which	513 (29.1)
Domestic assistance	289 (56.8)
Domestic assistance and personal care	161 (31.6)
Personal care	59 (11.6)
**Patient was an informal caregiver (*n*** **= 1842, 8 missing)**	
No	1325 (71.9)
Yes ^d^	505 (27.4)
Does not know	12 (0.7)

^a ^All patients answered the ‘What matters most’ and ‘Why it matters’ questions. Demographic data on some patients were missing as can be seen in the table

^b ^Unless otherwise indicated, data are presented as No. (%) of patients

^c^1 month before admission

^d ^Informal caregiver for child(ren), partner, parent(s), friend(s), acquaintance(s), animal(s)

### What matters most to patients and why?

The coding framework included twelve categories (*health, getting home, symptom relief, functioning, medical issues, hospital experience, patient values, reassurance, possessions, emotions, urgency,* and *other)*. These categories were divided into 38 sub-categories (e.g. ‘symptom relief’ was divided into pain, dyspnoea, fatigue, nausea). Table S1 in the Supplementary Material shows the categories and sub-categories, illustrated by explanations and quotes.

**Fig. 2:**
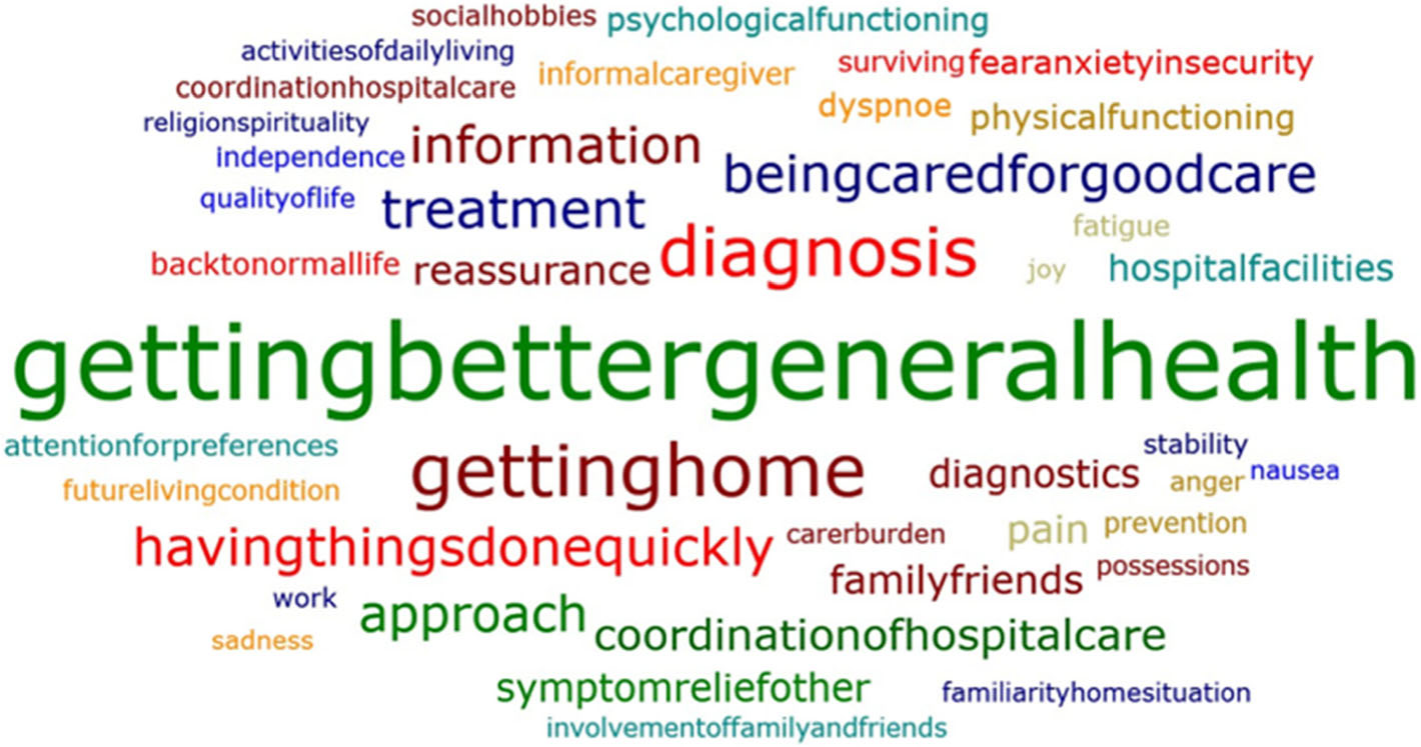
Word cloud of ‘what matters most’

To most answers, two to four categories were assigned. Of all patients, 29.6% answered that being in *good health* or *getting better* was most important at that moment, 17.4% said they wanted to *go home* and 16.1% considered *knowing the diagnosis* was most important. These categories were assigned notably more often than others (see Fig. 2 and Table S2 in the Supplementary Material).

**Fig. 3:**
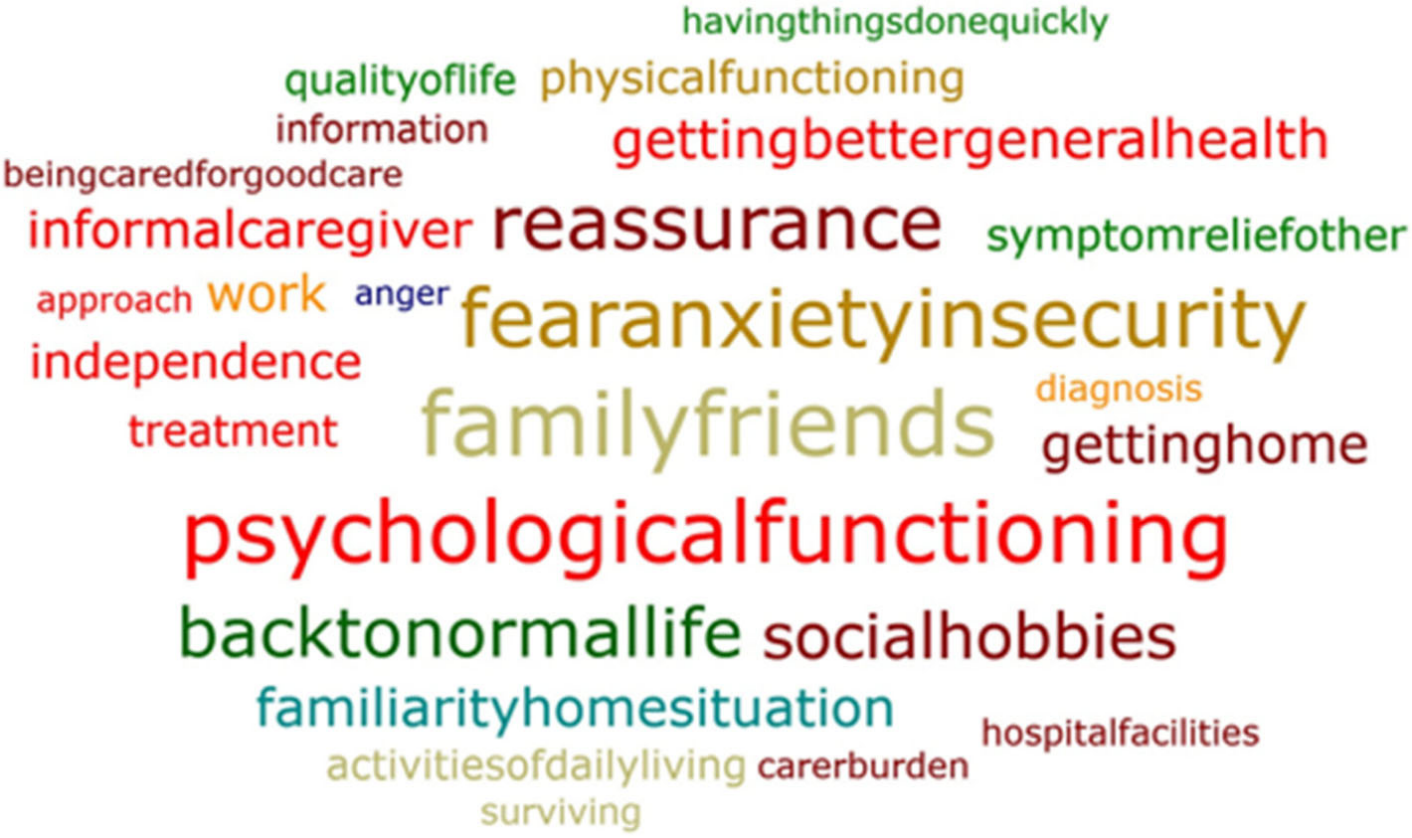
Word cloud of ‘Why does this matters most’

Compared to the answers to the ‘what matters most?’ question, the answers to ‘why does this matters most?’ showed a broader range of categories, with no clear top three (see Fig. 3). *Health* was mentioned less often as an underlying reason compared to the ′what matters′ question (Supplementary Material: Table S3). Many issues were mentioned by comparable numbers of patients (e.g. *family and friends (11.8%), psychological functioning (11.2%), fear, anxiety* and *insecurity (10.4%))*.

### Combined occurrence of *what* matters and *why*

Underlying reasons for ‘what mattered most?’ were given when asked ‘why this mattered most?’. Analysis of answers to the ‘what matters most?’ and ‘why?’ questions, revealed combinations of answers that occurred frequently together. Illustrations of apparent combinations observed in the top three ‘what matters most?’ categories are shown below.

### Getting better

Most patients wanted to get better *to be reunited with their loved ones* (usually partner or children, sometimes friends or other family members): “I miss my two-year-old son and sense that he is missing me a lot too. I want to get better so I can take care of my son and to have the energy to do fun things with him.” (Female, age-group 31–35 years, The Netherlands), “To get rid of my alcohol problem. It is important because it is destroying me and my family.” (F, 56-60Y, Denmark), “It’s important for me to recover as my children and grandchildren depend on me for money.” (M, 61-65Y, United Kingdom) Other patients wanted to get better *to get back to their normal life*: “That I will be able to do everything I feel like again.” (M, 71-75Y, The Netherlands).

### Getting home

Most patients mentioned the *familiarity of the home situation*, their role as an *informal caregiver* or *relationships* as the main reason to strive for a return to home. Examples include: “I feel better at home, having your own stuff around.” (M, 71-75Y, The Netherlands), “At home I feel most comfortable, they have no dark beer here.” (M, 86-90Y, The Netherlands), “To get home to my wife and our 3-year old daughter. My wife is expecting, I just cannot bear the thought of her giving birth without me.” (M, 41-45Y, Denmark), “My husband is 80. It is more difficult for him to visit me in hospital now.” (F, 67-80Y, United kingdom), “Wish to get home to my daughter- in-law’s 50th birthday on Friday.” (M, 66-70Y, Denmark).

### Getting a diagnosis

The wish for an established *diagnosis* was most often expressed in combination with *fear and insecurity*. Patients wanted *reassurance* and felt having a diagnosis would make them *function better psychologically*. “To know what is wrong for peace of mind.” (M, 46-50Y, The Netherlands), “I want to be able to do my own research or reading about the diagnosis.” (F, 66-70Y, United Kingdom), “It is unsafe to be sent home without clarification.” (F, 31-35Y, Denmark), “That I get my diabetes management optimised, even though I’m admitted with a COPD exacerbation. I’m scared that my legs will need amputating and then I can’t live in my apartment and keep my 11-year-old dog anymore.” (M, 51-55Y, Denmark), “To find peace of mind and closure. I’m afraid of Alzheimer’s and aging, it is affecting work.” (F, 56-60Y, Ireland).

### Patient perspective: does your doctor know?

More than half of all patients (51.9%) felt their treating doctor did not know what mattered to them most. Of this group, some patients (21.3%) reported to not have seen a doctor yet. Other reasons included “it did not come up in the conversation”, “the doctor does not need to know”, “there was no chance or no reason to tell”, or “the doctor did not listen” (Table 2*)*.

**Table 2:** Patient perspective: does your doctor know what matters most to you?

Does your doctor know what matters most?	No (%)
Yes	886 (48.1)
No ^a^	861 (46.7)
No, but someone else from the health care professional team knows ^a, b^	96 (5.2)
Did not speak to the doctor yet	202 (21.3)
Doctor does not need to know	165 (17.4)
The doctor did not listen	45 (4.7)
Other reason	538 (56.6)
Did not talk about it ^c^	219
No reason to tell ^d^	67
No chance to tell ^e^	53
Other reason ^f^	44
Unknown	162

^a ^When patient felt the doctor did not know, a follow-up question was asked

^b ^(e.g. nurse, physiotherapist, etc.)

^c ^I.e. doctor did not ask (78), patient did not tell (40), not covered in conversation (101)

^d ^I.e. assuming the doctor knows (29), expectations already met (7), not relevant (19), too early to get answers (5), a nurse knows (7)

^e ^I.e. insufficient continuity of care (7), doctor was too busy (28), do not know who my doctor is (8), afraid to tell (5), doctor did not care (5)

^f^ I.e. does not remember (4), other reason (35), does not know (5)

### Subgroup analysis

Women more frequently considered the way that they were *approached* by healthcare staff (e.g. a kind approach, personal attention, honesty, openness, feeling supported, being treated with respect and dignity) as most important (12.2% of women, 5.6% of men). We found no major differences in both ‘*what* matters most?’ and ‘*why?’* between different age groups (18–40, 41–70, 71+), patients with different length of stay (≤6 and > 6 h), and those who felt that the doctor knew (or not) (Supplementary Material: Table S4). In Asian countries we found a relatively high percentage of patients mentioning *getting better/ good health* as being most important (47.8–65.8% in Asian countries, 18.3–39.0% in Western countries). Patients in Singapore mentioned their *work* as the reason *why* things mattered more often than patients in other countries (17.1% and ≤ 7.2% respectively) (Supplementary Material: Table S5).

## Discussion

In this study 1850 patients admitted acutely to sixty-six hospitals in seven countries were asked what mattered most to them and why. Irrespective of the country, disease- and care-related issues were predominant in reply to the ‘what matters most?’ question: getting better, knowing the diagnosis and being able to go home. This is in line with the main function of an acute hospital admission and the motivation and focus of clinicians: diagnosing, treating and timely discharge [33]. However, when asked why they answered the way they did, patients provided more personal answers, often mentioning relationships and psychological well-being. Whereas many patients mentioned the same issues to the question ‘what matters most to you?’, the underlying reasons as to ‘why is this important?’ differed significantly from patient to patient. This probably reflects the heterogeneity of acutely admitted patients with regards to morbidity, baseline characteristics, culture, health, socio-economic status and phases of their lives. It demonstrates the challenges of providing patient-centred care without discussing what is most important with each individual patient.

Although certain combinations of *what matters?* and *why?* were more common than others, and some categories were mentioned more frequently within certain subgroups of patients, individual priorities are not predictable. Knowing what matters to each individual patient is key [34–36] because, as our data shows, it is a reflection of personal goals and preferences.

A large group of patients felt the treating doctor was unaware of what mattered most to them, partly because it did not come up during the consultation. Doctor-patient communication is crucial to the doctor-patient relationship [37], and essential in delivering high quality care, since the priorities of doctors and patients can differ [38]. It is conceivable that doctors focus mainly on diagnosing and treating the underlying medical condition. However, since the data represents the perception of patients, it is also possible that doctors do know what matters most, without the patient consciously realizing this. As the feeling of being heard and understood is essential in the process of patient-centred decision-making [39], it is recommended to have explicit conversations about what matters most and why, even if the doctor believes they already know this. Feeling heard and understood is known to alleviate suffering [40, 41], reinforce dignity [42, 43] and is one of the key factors in patient reported quality of care [44, 45]. It could help making patients feel that doctors see them as a person instead of a disease to be treated.

In healthcare settings with limited time per patient, these two simple questions (‘what matters most to you?’ and ‘why?’) may be a feasible way to quickly get to know the person behind the patient. The conversation will give insight into the personal situation of the patient, stimulate patient involvement and ultimately could facilitate more patient-centred care [46]. Having these conversations early in the admission will help set the agenda and design a tailored care plan [8, 47, 48].

### Strengths and limitations

The flash mob research design enabled us to include many patients within a short timeframe in seven different countries and 66 hospitals, across cities, towns and rural areas. It provided data from a large heterogeneous patient population representative of the wide diversity of acutely admitted patients. There were no missing data in the main questions. The scale of the study has enabled us to create awareness among many healthcare providers and patients. Lastly, we developed a new conceptual framework based on multiple perspectives using an iterative process. Answers were coded by both a medical doctor and psychologist, which ensured capturing the medical as well as the psychological component. The framework is comprehensive and suitable for the broad concept of ‘what matters most?’ and ‘why?’. Therefore, we believe the framework will be suitable for use in other patient groups and settings as well.

The results of our study need to be interpreted in the light of a few limitations. Firstly, answers from patients might have been paraphrased, which may have simplified patient answers. Secondly, due to the large number of interviewers, it is possible that there were differences in interview styles. However, as there were only two, highly standardized main questions, we believe this would not have had a significant influence on our results.

Future research might focus on how ‘what matters most’ to patients might change over the course of a hospital admission. Although no large differences were found between patients that had only spent up to 6 h in hospital and those in hospital from six to 24 h, we do not know whether the findings are representative for what matters most to patients in later phases of their admission. Furthermore, it would be interesting to conduct a study where both the patient, the doctor and all other professionals in the healthcare team are interviewed about what matters most to the patient in order to compare and align their views.

## Conclusions

Patients most frequently mentioned the importance of getting better, having a diagnosis and going home in the first 24 h of an admission. ‘Why’ this matters is strongly determined by each individual patient and often goes well beyond the medical targets of healthcare professionals. When asking for the patient perspective, a large group of patients felt the treating doctor did not know what mattered to them. Explicitly asking ‘what matters most?’ and especially ‘why?’, may help the healthcare team to obtain a more holistic picture and to see the person behind the patient. Having conversations regarding what is important to the patient should assist with the design of a personalized care plan and will help the patient to feel heard, which positively effects the patient satisfaction, health outcomes and the overall quality of care.

## Supplementary Information

**Additional file 1: Figure S1.** Developmental process of framework. **Table S1.** Framework for coding. **Table S2.** Top ten answers to the question ‘what matters most’. **Table S3.** Top ten answers to the question ‘why is this important’. **Table S4.** Differences in what matters and why between sex, age groups, length of stay and if patients feel the doctor knows what matters or not. **Table S5.** Differences in what matters and why to patients between countries. List of local collaborators.

## Data Availability

The anonymized dataset is available on reasonable request after approval of the corresponding author.

## Abbreviations

NTR: Netherlands Trial Register
AMUs: Acute medical units
