# Correction to: Understanding what matters most to patients in acute care in seven countries, using the flash mob study design

**DOI:** 10.1186/s12913-021-06580-4

**Published:** 2021-06-18

**Authors:** Eva S. van den Ende, Bo Schouten, Marjolein N. T. Kremers, Tim Cooksley, Chris P. Subbe, Immo Weichert, Louise S. van Galen, Harm R. Haak, John Kellett, Jelmer Alsma, Victoria Siegrist, Mark Holland, Erika F. Christensen, Colin A. Graham, Ling Yan LEUNG, Line E. Laugesen, Hanneke Merten, Fraz Mir, Rachel M. Kidney, Mikkel Brabrand, Prabath W. B. Nanayakkara, Christian H. Nickel, on behalf of all local collaborators

**Affiliations:** 1 Section of Acute Medicine, Department of Internal Medicine, Amsterdam Public Health research institute, Amsterdam University Medical Center, location VU University Medical Center, De Boelelaan 1117, 1081 HV Amsterdam, ZH The Netherlands; 2 Department of Public and Occupational Health, Amsterdam Public Health research institute, Amsterdam University Medical Center, location VU University Medical Center, the Ende et al. BMC Health Services Research (2021) 21:474 , Amsterdam, the Netherlands; 3 Department of Health Services Research, CAPHRI School for Public Health and Primary Care, Aging and Long Term Care, Maastricht University, Maastricht, the Netherlands; 4 Department of Internal Medicine, Máxima MC, Veldhoven/Eindhoven, The Netherlands; 5 Department of Acute Medicine, University Hospital of South Manchester, Manchester, UK; 6 Department of Acute Medicine, Ysbyty Gwynedd Hospital, Bangor, UK; 7 School of Medical Sciences, Bangor University, Bangor, UK; 8 Department of Acute Medicine, Ipswich Hospital, East Suffolk and North Essex NHS Foundation Trust Ipswich Hospital, Ipswich, UK; 9 Department of Internal Medicine, Division of General Internal Medicine, Maastricht University Medical Center, Maastricht, The Netherlands; 10 Department of Emergency Medicine, Hospital of South West Jutland, Esbjerg, Denmark; 11 Department of Internal Medicine, Erasmus University Medical Center, Rotterdam, the Netherlands; 12 Department of Cognitive and Decision Sciences, University of Basel, Basel, Switzerland; 13 Department of Emergency Medicine, University Hospital Basel, Basel, Switzerland; 14 Section of Acute Medicine, Department of Internal Medicine, Salford Royal NHS Foundation Trust, Salford, UK; 15 Center for Prehospital and Emergency Research, Clinic of Internal and Emergency Medicine, Aalborg University Hospital and Aalborg University, Aalborg, Denmark; 16 Institute of Clinical Medicine, Aalborg University Hospital and Aalborg University, Aalborg, Denmark; 17 Department of Emergency Medicine, Chinese University of Hong Kong, Hong Kong, Hong Kong; 18 Department of Medicine, Addenbrooke’s Hospital, Cambridge, UK; 19 Department of Internal Medicine, St. James’s Hospital, Dublin, Ireland; 20 Department of Emergency Medicine, Odense University Hospital, Odense, Denmark

**Correction to: BMC Health Serv Res 21, 474 (2021)**

**https://doi.org/10.1186/s12913-021-06459-4**

Following publication of the original article [1], the authors identified an error in the author name of Ling Yan LEUNG.

The incorrect author name is: L. E. U. N. G. Ling Yan

The correct author name is: Ling Yan LEUNG

The author group has been updated above and the original article [1] has been corrected.
