# Supplementary material for: Understanding what matters most to patients in acute care in seven countries, using the flash mob study design
Source: BMC Health Serv Res. 2021 May 19;21:474. doi: 10.1186/s12913-021-06459-4 (PMC8132421; doi:10.1186/s12913-021-06459-4)
Supplement: Supplementary file 1 — Additional file 1: Figure S1. Developmental process of framework. Table S1. Framework for coding. Table S2. Top ten answers to the question ‘what matters most’. Table S3. Top ten answers to the question ‘why is this important’. Table S4. Differences in what matters and why between sex, age groups, length of stay and if patients feel the doctor knows what matters or not. Table S5. Differences in what matters and why to patients between countries. List of local collaborators. [file 12913_2021_6459_MOESM1_ESM.docx]

**Supplementary Material**

Understanding what matters most to patients in acute care in seven countries, using the flash mob study design

Eva S. van den Ende, MD^1 a^; Bo Schouten, MSc^2 a^; Marjolein N. T. Kremers, MD^3 4^; Tim Cooksley, MD^5^; Chris P. Subbe, MD^6 7^; Immo Weichert, MD^8^; Louise S. van Galen, MD, PhD^1^; Harm R. Haak, MD PhD^3 4 9^; John Kellett, MD^10^; Jelmer Alsma, MD, PhD^11^; Victoria Siegrist, MSc, PhD^12 13^; Mark Holland, MD^14^; Erika F. Christensen, MD, PhD^16 17^; Colin A Graham, MD, MPH, FHKCEM^18^; LEUNG Ling Yan, BSc, PhD^18^; Line E. Laugesen^10^; Hanneke Merten, MSc PhD^2^; Fraz Mir, MD^19^; Rachel M Kidney, MD^20^; Mikkel Brabrand, MD, PhD^10 21 b^; Prabath W.B. Nanayakkara, MD, PhD, FRCP^1 b^; Christian H. Nickel, MD, PhD^13 b^; on behalf of all local collaborators

*^1^* Section of Acute Medicine, Department of Internal Medicine, Amsterdam Public Health research institute, Amsterdam University Medical Center, location VU University Medical Center, Amsterdam, the Netherlands

*^2^* Department of Public and Occupational Health, Amsterdam Public Health research institute, Amsterdam University Medical Center, location VU University Medical Center, Amsterdam, the Netherlands

*^3^* Department of Health Services Research, CAPHRI School for Public Health and Primary Care, Aging and Long Term Care, Maastricht University, Maastricht, the Netherlands

^4^ Department of Internal Medicine, Máxima MC, Veldhoven/Eindhoven, the Netherlands

^5^ Department of Acute Medicine, University Hospital of South Manchester, Manchester, United Kingdom

^6^ Department of Acute Medicine, Ysbyty Gwynedd Hospital, Bangor, United Kingdom

^7^ School of Medical Sciences, Bangor University, Bangor, United Kingdom

^8^ Department of Acute Medicine, Ipswich Hospital, East Suffolk and North Essex NHS Foundation Trust, Ipswich, United Kingdom

^9^ Department of Internal Medicine, Division of General Internal Medicine, Maastricht University Medical Center, Maastricht, the Netherlands

^10^ Department of Emergency Medicine, Hospital of South West Jutland, Esbjerg, Denmark

^11^ Department of Internal Medicine, Erasmus University Medical Center, Rotterdam, the Netherlands

^12^ Department of Cognitive and Decision Sciences, University of Basel, Basel, Switzerland

^13^ Department of Emergency Medicine, University Hospital Basel, Basel, Switzerland

^14^ Section of Acute Medicine, Department of Internal Medicine, Salford Royal NHS Foundation Trust, Salford, United Kingdom

^15^ Faculty of Health and Welling, University of Bolton, Bolton, United Kingdom

^16^ Center for Prehospital and Emergency Research, Clinic of Internal and Emergency Medicine, Aalborg University Hospital, Aalborg, Denmark

^17^ Institute of Clinical Medicine, Aalborg University, Aalborg, Denmark

^18^ Department of Emergency Medicine, Chinese University of Hong Kong, Hong Kong

^19^ Department of Medicine, Addenbrooke’s Hospital, Cambridge, United Kingdom ^20^ Department of Internal Medicine, St. James's Hospital, Dublin, Ireland ^21^ Department of Emergency Medicine, Odense University Hospital, Odense, Denmark

^a^ Authors contributed equally to this work

^b^ Authors equally supervised the work

**Correspondence to:** Prabath W.B. Nanayakkara, MD, PhD, FRCP, Section of Acute Medicine, Department of Internal Medicine, Amsterdam University Medical Center, location VU University Medical Center, Amsterdam, the Netherlands. De Boelelaan 1117, 1081 HV Amsterdam, ZH 1D57.2

[p.nanayakkara@amsterdamumc.nl](mailto:p.nanayakkara@amsterdamumc.nl) (ORCID 0000-0002-1555-3682)

**Table of contents**

**Figure S1.** Developmental process of framework

**Table S1.** Framework for coding

**Table S2.** Top ten answers to the question ‘what matters most’

**Table S3.** Top ten answers to the question ‘why is this important’

**Table S4.** Differences in what matters and why between sex, age groups, length of stay and if patients feel the doctor knows what matters or not

**Table S5.** Differences in what matters and why to patients between countries

List of local collaborators

**Figure S1. Developmental process of framework**

^a^ EE, MK (medical doctors) and BS, HM (psychologists)

^b^ Jenkinson C, Coulter A, Bruster S, Richards N, Chandola T. Patients' experiences and satisfaction with health care: results of a questionnaire study of specific aspects of care. Quality & safety in health care. 2002;11(4):335-9.

**Table S1. Framework for coding**

| Main Theme | Sub-Theme | EXPLANATION | Quotes |
| --- | --- | --- | --- |
| Health | Getting better / good health | Getting well, general concept of health | *“My health”, “To get better”, “To recover from what has happened”, “Health improvement”, “To be as healthy as possible and to stay heathy”, “To get healthier: I suffer from pneumonia, and something in my lungs that is not right”, “Being healthy and a healthy life”, “That the blood pressure goes down”.* |
|  | Stability | Health remains stable, not to deteriorate | *“No further decline of heart and kidneys”, “To maintain a reasonable standard of health”, “I'm not going to be cured but….”* |
|  | Surviving | Not dying, continue living, to be able to live | *“Just to continue living”, “I want to survive”, “To stay alive”* |
| Getting home | Getting home | To go home and/or to be discharged and leave the hospital | *“To be allowed to go home”, “Return to residence and workplace (Paris)”, “To be discharged as soon as possible. Did not wanted to be admitted and wants to go home at least before noon”.* |
|  | Familiarity of the home situation | Wanting to be at home, feeling better at home, having your own stuff around | *“I prefer to be at home”, “Home feels more pleasant”, “At home it is nicer than in the hospital”, “I want my own things around me”, “I like it way better at home, familiar situation. There I have my medication in my own management, so I know I take them correctly. Also, I have privacy at home (alone instead of a room for four people).”* |
|  | Future living condition | Help at home, having a place to go to, moving to a different place, a roof over your head | *“To get the needed care at home or at the nursing home”, “A new home, shelter. I have no roof over my head”, “That the home caregivers are at home with me for almost the entire day, help me with everything”, “I want to stay out of prison longer”.* |
| Symptom relief | Pain | Being pain-free | *“Living without pain”, “The pain is not bearable right now”, “When you have been in pain a long time it grows on you. Feels it's important for no one to be in pain if possible”.* |
|  | Nausea | Relief of nausea | *“I have a gastric carcinoma, want to get rid of de nausea”, “That I get rid of the nausea and can eat again”.* |
|  | Dyspnoea | Relief of shortness of breath | *“To breath more easily”, “I am worried about breathing and not being able to breath”* |
|  | Fatigue | Rest, sleep quality | *“To be able to sleep again”, “Fatigue is very annoying and because of this I cannot do much”, “Just to lie down and rest”.* |
|  | Other | Relief of all other symptoms, complaints and discomfort | *“I am hungry and would like to eat something”, “Getting rid of my atrial flutter”, “That my complaints and fever are taken care of”, “Not being thirsty anymore”, “My vertigo to pass”, “Getting my symptoms managed”, “To make me comfortable”.* |
| Functioning | Social & hobbies | I.e. religious activities, cycling, playing cards, vacation | *“I love gardening”, “I want to be able to go to football match”, “I still want to do a lot in life”, “I want to be able to go bicycling with my wife again. I had a bicycle shop previously. Bicycling is my hobby and life's work”, “I want to have the energy for activities, like reading, shopping groceries and go to concerts”.* |
|  | Psychological | LONG TERM behaviour, coping skills, and overall mental health | *“I want peace and clarity on what the future will look like”, “Happiness”, “To become the old me again, that I used to be”, “I wish I never had COPD a self-made disease. Feels it is my fault, caused by being selfish”, “To have a healthy mind”.* |
|  | Physical | I.e. regaining strength, Gaining weight, Physical condition, walking, be able to move arm/legs again, physical capabilities, physical appearance | *“To sit up for another twenty minutes”, “To be able to walk again”, “To be able to see with both eyes”, “To recuperate and regain strength”, “To be able to speak clearly again. Admitted after rehabilitation. Since yesterday difficulties to speak”.* |
|  | Informal caregiving | Patient takes care of people or animals (NOT if patient HAS an informal caregiver) | *“My younger brother has low IQ and unable to take care of himself”, “That I can't be there for my kids right now and make them anxious”, “That someone takes care of my husband while I'm in hospital”, “It's important for me to recover as my children and grandchildren depend on me for money and help with ‘do it yourself’, advice etc.”, “That someone can take care of the dog at home”.* |
|  | Activities of daily living | Activities/skills performed on a daily basis fundamental for basic needs, i.e. household chores, personal hygiene, etc. | *“That I can get out of bed independently and do things myself again”, “I'd like to restart activities at home”, “To be able to function at home”, “I want to do the grocery shopping and housekeeping”.* |
|  | Work | I.e. study, have to get back to work | *“A lot of problems, but the main one is that I can't work currently”, “School/career”, “I’m missing time at work due to recent admission”.* |
|  | Back to normal life | Return to (parts of) their normal life, i.e. their life as it was before the acute hospital admission | *“I want to live a normal life, without my abdomen bothering me”, “That I can continue driving the car”, “To be able to continue living like I used to do”.* |
| Medical ISSUES | Diagnosis | ASSUMING THIS IS STILL UNKNOWN BY HEALTHCARE TEAM: Diagnosis, prognosis, finding out what patient is suffering from | *“Clarity about what is wrong and what the consequences of the disease are for the chemotherapy treatment”, “That they find what is wrong with me”.* |
|  | Testing (diagnostics) | Test result, getting medical test, physical examination (if NO MORE tests: also add “other”) | *“I want proper diagnostics”, “Even if it's not sure, I would like to know what my complaints may indicate and what the diagnosis might be”, “I want the cause of symptoms being examined”.* |
|  | Treatment | Getting the right treatment, treatment plan, results of treatment or procedures, timing of treatment (if STOP treatment: also add “other”) | *“The removal of my kidney drain and right kidney”, “I want non-invasive treatment”, “To have better blood pressure control”, “I came here for my bladder problem, maybe to take out my prostate; to get treated”.* |
|  | Prevention | to prevent a similar health condition and/or deterioration in the future | *“I want to know how to prevent these symptoms in the future”, “I want to prevent amputation as that would impede my abilities to take care of household”.* |
| Hospital experience | Receiving information | ASSUMING THIS DIAGNOSIS IS KNOWN BY HEALTHCARE TEAM: Getting clear information, getting explanation about diagnosis, being well informed | *“Communication is key. When in hospital you in-trust other people to make the best decision on your behalf, therefore being kept in the loop reduces worry”, “To be shown what will be the next step”, “I want to know how long I'll have to stay here”, “To find out what possibilities there are for recovery”.* |
|  | Coordination of hospital care | I.e. COMMUNICATION BETWEEN PROFESSIONALS, waiting times, hospital processes, familiar physician, teamwork of staff | *“It means a lot to me that it is easy to transfer information from general practitioner to the hospital”, “Less waiting time”, “I would like to have the same physician throughout. I keep seeing different ones. I'd like to have a contact person”.* |
|  | Approach by healthcare staff | I.e. COMMUNICATION WITH PATIENT. (Kind) approach, personal attention, patient centeredness, honesty, openness, feeling supported, professional listens, has time, treated with respect and dignity. | *“Empathy, I have been in hospital before and remember the empathy of the staff”, “To feel recognized and noticed”, “A secure and safe stay, with mutual respect”, “That the staff recognizes and respects my boundaries”, “It is important that I’m taken seriously, in what I say”, “A good welcome”.* |
|  | Attention for preferences | RELATED TO TREATMENT: shared decision making, Involvement in decisions about treatment and care. | *“I am plagued by old injuries, so I know best how my body works. To talk openly is important”, “I have chosen to do without the treatment because of side effects”, “I do not worry because I feel involved”.* |
|  | Involvement of family and friends | Information and decision-making in presence of family | *“I want to be able to clearly explain to my family what is wrong and my treatment plan. I don't want to seem 'dumb'”, “It's important to me that my family don't needlessly worry. I don't want them in emotional distress and would prefer they knew what to expect in terms of outcome”,” The care is important both to me and my relatives”.* |
|  | Facilities | I.e. hospital beds, -rooms, -food, coffee, tranquillity, privacy, circumstances stay, visiting hours, eating, smoking, hospital environment | *“Getting an appropriate mattress (in hospital), making it very soar for back”, “That I am allowed to have my good friend by my side”, “I have not slept all night because of the noise from appliances”, “Good food and quietness to get sleep”, “That there is good coffee”, “It's too hot, can someone cool down the place?”.* |
|  | Being cared for / good care | I.e. getting the help that is needed, not being discharged too soon, getting better before discharge, hospital admission, trust in professionals, receiving good care and treatment, adequacy, competence, expertise, care at the ED, trust. | *“It is degrading to be as vulnerable as you are when you are ill. Therefore, it is important to me to be cared for”, “That I'm being taken care of”, “That somebody takes care of me and my problem”, “The care at the ED”, “To receive good hospital care, even if it is busy”.* |
| PATIENT Values | Family and/or friends | I.e. partner, family, friends, pets | *“Family, Child, Son and Daughters. I'd rather be in a different hospital. I don't know anyone here”, “What's extremely important to me is visiting my family in a different canton on Thursday”, “Lost my partner. Childhood dreams are gone. To experience more love”.* |
|  | Independence | I.e. being independent from others, freedom | *“Want to keep my independence”, “Regain autonomy”, “I feel a bit trapped, my freedom is limited”, “Do not like to be dependent on others to be taken to the hospital, as I do not live nearby”.* |
|  | Carer burden | The potential burden the patient and the health condition could put on the carers (i.e. family, friends, etc.) | *“I want to go come Saturday early noon because my kids will pick me up. At night it will be inconvenient for them”, “I don’t want to put too much carer burden on my partner. To give her the least possible burden”.* |
|  | Quality of life | I.e. enjoying life,, end of life care, expectations about the future, wanting to die | *“To improve quality of life. Now I have no quality of life”, “I would like the optimal quality of life that can be reached for my age (the level of a couple of months ago, before the complaints started)“.* |
|  | Religion & spirituality | Religious and spiritual beliefs | *“Receiving the right care in the spiritual field”, “God, I’m a pastor in Pakistan”, “That god helps me with this”.* |
| Reassurance |  | Desire to be reassured by professionals, i.e. wanting clarity, certainty, feeling ensured that everything will be fine | *“Older people need safety to function”, “That everything is going to be fine”, “Being kept in the loop, I am an ex-military, it’s the way I was trained and it gives me reassurance”.* |
| Possessions |  | I.e. finances, hospital bills, daily expenditure, personal belongings | *“I have been admitted yesterday, still waiting. I think it is a loss of money, as I’m only here for examinations that have a longer waiting time at the outpatient clinic”, “I worry making too little money and not being able to take care of the family”, “Money - hospital bills and daily expenditure”* |
| Emotions | Negative | SHORT TERM expression of mood and/or feelings, i.e. fear, anxiety, insecurity, anger, disappointment, frustration, sadness, despair | *“Medication being changed on me for no real reason. It's uncomfortable and unfamiliar to me, especially since medication prescribed by GP who has cared for me for 4 years”, “Because the staff react right away I become insecure because I sense that I am an acute patient”, “I'm nervous that my cancer operation will be postponed”, “Anxiety since the cancer diagnosis”. “I am just waiting for nothing to happen. Because one can get the feeling of being put on a shelf and just wait for information about ones’ course if no one speaks to you”, “I was very angry with the nurse”, “Very sad to hear the diagnosis”, “I want to know the future for my wife. She is from Thailand, and if I die, she has to leave the country within three months. I feel sorry for her”.* |
|  | Positive | SHORT TERM expression of mood and/or feelings, i.e. joy | *“I love to enjoy a cup of good coffee”, “So happy to finally have a diagnosis”.* |
| Having things done quickly |  | Quickly, immediate | *“To be discharged as soon as possible. Did not wanted to be admitted and wants to go home at least before noon”, “Being assessed quickly, that my treatment starts immediately”.* |
| Other |  | Politics, etc. | *“To allow others to get access to treatment”, “Nothing really truly matters to me anymore. I have lived for many years almost 90”, “Politics”, “The Brexit and remaining in Europe”.* |

**Table S2. Ten most frequent answers to the question ‘what matters most’**

| What matters most to you at the moment? - Top 10 answers | n | % |
| --- | --- | --- |
| Getting better / good health^a^ | 547 | 29.6% |
| Getting home | 322 | 17.4% |
| Knowing the diagnosis | 298 | 16.1% |
| Treatment | 192 | 10.4% |
| Having things done quickly | 185 | 10.0% |
| Being cared for / receiving good care | 184 | 9.9% |
| Receiving information | 179 | 9.7% |
| Approach (attitude of healthcare staff) | 169 | 9.1% |
| Coordination of hospital care | 127 | 6.9% |
| Reassurance | 101 | 5.5% |

*n = number of patients
%= percentage of all included patients (N=1850)
^a^ obtaining good health or staying in good health*

**Table S3. Ten most frequent answers to the question ‘why is this important’**

| Why it matters - Top 10 answers | n | % |
| --- | --- | --- |
| Family and/or friends | 218 | 11.8% |
| Psychological functioning^a^ | 208 | 11.2% |
| Fear/ anxiety/ insecurity | 192 | 10.4% |
| Reassurance | 173 | 9.4% |
| Back to normal life | 150 | 8.1% |
| Social activities / hobbies | 132 | 7.1% |
| Getting better / general health | 104 | 5.6% |
| Familiarity of home situation^b^ | 101 | 5.5% |
| Getting home | 98 | 5.3% |
| Role as an informal caregiver | 95 | 5.1% |
| Work | 92 | 5.0% |

*n = number of patients
%= percentage of all included patients (N=1850)
^a^ e.g. coping skills, and overall mental health
^b^ e.g. Wanting to be at home, feeling better at home, having your own stuff around*

**Table S4. Differences in what matters most and why between: sex, age groups, length of stay and if patients feel the doctor knows what matters or not.**
 **WHAT matters most**

|  | Doctor DOES know (n=886) | Doctor does NOT know (n=957) |
| --- | --- | --- |
| 1 | Getting better / good health n=258 (29.1%) | Getting better / good health n=290 (30.3%) |
| 2 | Getting home n=157 (17.7%) | Getting home n=163 (17.0%) |
| 3 | Knowing the diagnosis n=154 (17.4%) | Knowing the diagnosis n=143 (14.9%) |
| 4 | Treatment n=108 (12.2%) | Receiving information n=103 (10.7%) |
| 5 | Being cared for / good care n=89 (10.0%) | Having things done quickly n=102 (10.7%) |

|  | Male (n=918) | Female (n=918) |
| --- | --- | --- |
| 1 | Getting better / good health n=282 (30.7%) | Getting better / good health n=264 (28.8%) |
| 2 | Getting home n=163 (17.8%) | Getting home n=157 (17.1%) |
| 3 | Knowing the diagnosis n=157 (17.1%) | Knowing the diagnosis n=141 (15.4%) |
| 4 | Having things done quickly n=103 (11.2%) | Approach n=112 (12.2%) |
| 5 | Treatment n=101 (11.0%) | Receiving information n=97 (10.6%) |

|  | <6 hours after admission (n=688) | ≥6 hours after admission (n=1152) |
| --- | --- | --- |
| 1 | Getting better / good health n=189 (27.5%) | Getting better / good health n=357 (31.0%) |
| 2 | Knowing the diagnosis n=133 (19.3%) | Getting home n=227 (19.7%) |
| 3 | Getting home n=94 (13.7%) | Knowing the diagnosis n=166 (14.4%) |
| 4 | Approach n=76 (10.9%) | Receiving information n=119 (10.3%) |
| 5 | Treatment n=76 (10.9%) | Treatment n=118 (10.2%) |

|  | 18-40 years (n=195) | 41-70 years (n=799) | 71+ years (n=811) |
| --- | --- | --- | --- |
| 1 | Getting better / good health n=59 (30.3%) | Getting better / good health n=209 (26.2%) | Getting better / good health n=269 (33.2%) |
| 2 | Getting home n=40 (20.5%) | Knowing the diagnosis n=156 (19.5%) | Getting home n=151 (18.6%) |
| 3 | Knowing the diagnosis n=33 (16.9%) | Getting home n=126 (15.8%) | Knowing the diagnosis n=104 (12.8%) |
| 4 | Receiving information n=27 (13.8%) | Treatment n=99 (12.4%) | Being cared for / good care n=77 (9.5%) |
| 5 | Coordination of care n=25 (12.8%) | Having things done quickly n=93 (11.6%) | Treatment n=69 (8.5%) |

**WHY does this matter most**

|  | Doctor DOES know (n=886) | Doctor does NOT know (n=957) |
| --- | --- | --- |
| 1 | Fear / anxiety / insecurity n=91 (10.3%) | Family and/or friends n=136 (14.2%) |
| 2 | Psychological functioning n=91 (10.3%) | Psychological functioning n=116 (12.1%) |
| 3 | Family and/or friends n=81 (9.1%) | Fear / anxiety / insecurity n=100 (10.5%) |
| 4 | Reassurance n=78 (8.8%) | Reassurance n=94 (9.8%) |
| 5 | Back to normal life n=75 (8.5%) | Back to normal life n=75 (7.8%) |

|  | Man (n=918) | Female (n=918) |
| --- | --- | --- |
| 1 | Family and/or friends n=110 (12.0%) | Family and/or friends n=108 (11.8%) |
| 2 | Psychological functioning n=99 (10.8%) | Psychological functioning n=107 (11.7%) |
| 3 | Fear / anxiety / insecurity n=93 (10.1%) | Fear / anxiety / insecurity n=98 (10.7%) |
| 4 | Reassurance n=82 (8.9%) | Reassurance n=91 (9.9%) |
| 5 | Social activities & hobbies n=80 (8.7%) | Back to normal life n=73 (8.0%) |

|  | <6 hours after admission (n=688) | ≥6 hours after admission (n=1152) |
| --- | --- | --- |
| 1 | Reassurance n=69 (10.0%) | Family and/or friends n=155 (13.5%) |
| 2 | Fear / anxiety / insecurity n=66 (9.6%) | Psychological functioning n=142 (12.3%) |
| 3 | Psychological functioning n=65 (9.4%) | Fear / anxiety / insecurity n=126 (10.9%) |
| 4 | Family and/or friends n=62 (9.0%) | Reassurance n=103 (8.9%) |
| 5 | Getting home n=48 (7.0%) | Back to normal life n=103 (8.9%) |

|  | 18-40 years (n=195) | 41-70 years (n=799) | 71+ years (n=811) |
| --- | --- | --- | --- |
| 1 | Psychological functioning n=28 (14.4%) | Psychological functioning n=96 (12.0%) | Family and/or friends n=104 (12.8%) |
| 2 | Reassurance n=28 (14.4%) | Family and/or friends n=95 (11.9%) | Psychological functioning n=78 (9.6%) |
| 3 | Fear / anxiety / insecurity n=26 (13.3%) | Fear / anxiety / insecurity n=90 (11.3%) | Social activities & hobbies n=72 (8.9%) |
| 4 | Work n= 26 (13.3%) | Reassurance n=90 (11.3%) | Fear / anxiety / insecurity n=71 (8.8%) |
| 5 | Role as an informal care giver n=25 (12.8%) | Back to normal life n=75 (9.4%) | Back to normal life n=65 (8.0%) |

**Table S5. Differences in what matters and why to patients between countries**

**Top 5 what matters most**

|  | Denmark (n=654) | Netherlands (n=515) | UK (n=394) | Singapore (n=82) | Switzerland (n=91) | Ireland (n=41) | Hong Kong (n=73) |
| --- | --- | --- | --- | --- | --- | --- | --- |
| **1** | Knowing the diagnosis n=140 (21.4%) | Getting better / good health n=164 (31.8%) | Getting better / good health n=121 (30.7%) | Getting better / good health n=44 (47.8%) | Getting better / good health n=31 (34.1%) | Getting better / good health n=16 (39.0%) | Getting better / good health n=48 (65.8%) |
| **2** | Getting better / good health n=123 (18.3%) | Getting home n=105 (20.4%) | Getting home n=94 (23.9%) | Getting home n=11 (13.4%) | Getting home n=22 (24.2%) | Getting home n=8 (19.5%) | Getting home n=7 (9.6%) |
| **3** | Treatment n=97 (14.8%) | Knowing the diagnosis n=75 (14.6%) | Knowing the diagnosis n=55 (14.0%) | Having things done quickly n=7 (8.5%) | Knowing the diagnosis n=14 (15.4%) | Family and/or friends n=7 (17.1%) | Family and/or friends n=6 (8.2%) |
| **4** | Approach n=97 (14.8%) | Having things done quickly n=66 (12.8%) | Receiving information n=37 (9.4%) | Pain relief n=7 (8.5%) | Family and/or friends n=12 (13.2%) | Being cared for / quality of care n=5 (12.2%) | Knowing the diagnosis n=5 (6.8%) |
| **5** | Being cared for / quality of care n=93 (14.2%) | Treatment n=46 (8.9%) | Being cared for / quality of care n=36 (9.1%) | Treatment n=7 (8.5%) | Having things done quickly n=12 (13.2%) | Knowing the diagnosis n=5 (12.2%) | Familiarity home situation n=2 (2.7%) |

**Top 5 why is this important**

|  | Denmark (n=654) | Netherlands (n=515) | UK (n=394) | Singapore (n=82) | Switzerland (n=91) | Ireland (n=41) | Hong Kong (n=73) |
| --- | --- | --- | --- | --- | --- | --- | --- |
| **1** | Reassurance n=103 (15.7%) | Family and/or friends n=89 (17.3%) | Family and/or friends n=58 (14.7%) | Work n=14 (17.1%) | Social activities & Hobbies n=13 (14.3%) | Independence n=7 (17.1%) | Symptom relief other n=9 (12.3%) |
| **2** | Psychological functioning n=85 (13.0%) | Psychological functioning n=71 (13.8%) | Psychological functioning n=40 (10.2%) | Activities of daily living n=12 (14.6%) | Family and/or friends n=11 (12.1%) | Family and/or friends n=6 (14.6%) | Family and/or friends n=6 (8.2%) |
| **3** | Fear / anxiety / insecurity n=76 (11.6%) | Social activities & Hobbies n=66 (12.8%) | Fear / anxiety / insecurity n=37 (9.4%) | Role as an informal care giver n=11 (13.4%) | Getting home n=10 (11.0%) | Getting better / good health n=4 (9.8%) | Social and hobbies n=6 (8.2%) |
| **4** | Getting better / good health n=44 (6.7%) | Back to normal life n=59 (11.5%) | Back to normal life n=34 (8.6%) | Independence n=9 (11.0%) | Familiarity home situation n=9 (9.9%) | Quality of life n=4 (9.8%) | Back to normal life n=5 (6.8%) |
| **5** | Family and/or friends n=40 (6.1%) | Fear / anxiety / insecurity n=57 (11.1%) | Work n=28 (7.1%) | Symptom relief other n=9 (11.0%) | Having things done quickly n=8 (8.8%) | Reassurance n=4 (9.8%) | Getting home n=5 (6.8%) |

**List of local collaborators (local coordinators of participating centres)**

Vibe Maria Laden Nielsen, Aalborg University Hospital, Denmark; Karen Vestergaard Andersen, Aarhus University Hospital, Denmark; Hanne Nygaard, Bispebjerg Hospital, Denmark; Kasper Karmark Iversen, Herlev Hospital, Denmark; Martin Schultz, Herlev Hospital, Denmark; Peter Hallas, Holbæk Hospital, Denmark; Magnus Peter Brammer Kreiberg, Holbæk Hospital, Denmark; Line Emilie Laugesen, Hospital of South West Jutland, Esbjerg, Denmark; Anne Mette Green, Hospital of South West Jutland, Esbjerg, Denmark; Tanja Mose Kristensen, Hospital of South West Jutland, Esbjerg, Denmark; Helene Skjøt-Arkil, Hospital Sønderjylland, Aabenraa, Denmark; Hejdi Gamst-Jensen, Hvidovre Hospital, Denmark; Torbjørn Shields Thomsen, Hvidovre Hospital, Denmark; Camilla Dahl Nielsen, Kolding Hospital, Denmark; Kristian Møller Jensen, Kolding Hospital, Denmark; Søren Nygaard Hansen, Kolding Hospital, Denmark; Marc Ludwig, North Denmark Regional Hospital, Hjørring, Denmark; Henriette Sloth Høg, North Denmark Regional Hospital, Hjørring, Denmark; Dorthe Gaby Bove, North Zealand Hospital Hillerød, Denmark; Vibe Kristine Sommer Mikkelsen, North Zealand Hospital Hillerød, Denmark; Sune Laugesen, Odense University Hospital, Denmark; Nerma Todorovac, Odense University Hospital, Denmark; Stine Nørris Nielsen, Odense University Hospital, Denmark; Poul Petersen, Regional Hospital Herning, Denmark; Hanna Karstensen, Regional Hospital Herning, Denmark; Gitte Boier Tygesen, Regional Hospital Horsens, Denmark; Rasmus Aabling, Regional Hospital Horsens, Denmark; Lone Pedersen, Regional Hospital Randers, Denmark; Sef J L. W. Van Den Beuken, Regional Hospital Viborg, Denmark; Ditte Høgsgaard, Slagelse Hospital, Denmark; Thomas Christophersen, Svendborg Hospital, OUH, Denmark; Christina Smedegaard, Svendborg Hospital, OUH, Denmark; Mette Worsøe, Svendborg Hospital, OUH, Denmark; Marie-Laure M A Bouchy Jacobsson, Zealand University Hospital, Køge, Denmark; Le Elias Lyngholm, Zealand University Hospital, Køge, Denmark; Sara Fonager Lindholm, Zealand University Hospital, Køge, Denmark; JM van Pelt-Sprangers, Admiraal de Ruyter Ziekenhuis, The Netherlands; Ralph K.L. So, Albert Schweitzer Hospital, The Netherlands; Sander Anten, Alrijne Hospital, The Netherlands; Judith van den Besselaar, Alrijne Hospital, The Netherlands; Gerba Buunk, Amphia Hospital, The Netherlands; Lorenzo Romano, Amphia Hospital, The Netherlands; Daan Eeftick Schattenkerk, Amsterdam University Medical Center, location AMC, The Netherlands; Frits Holleman, Amsterdam University Medical Center, location AMC, The Netherlands; Rishi S. Nannan Panday, Amsterdam University Medical Center, location Vu Medical Center, The Netherlands; Sacha C. Rowling, Amsterdam University Medical Center, location Vu Medical Center, The Netherlands; Michiel Schinkel, Amsterdam University Medical Center, location Vu Medical Center, The Netherlands; Sophie van Benthum, Catharina Hospital, The Netherlands; S.J.J. Logtenberg, Diakonessenhuis Utrecht, The Netherlands; Esther M.G. Jacobs, Elkerliek Hospital, The Netherlands; Jelmer Alsma, Erasmus University Medical Center, The Netherlands; William Boogers, Erasmus University Medical Center, The Netherlands; Marlies Verhoeff, Gelre Hospitals, Apeldoorn and Zutphen, The Netherlands; Barbara V. van Munster, Gelre Hospitals, Apeldoorn and Zutphen, University Medical Center Groningen, The Netherlands; Emma Gans, Groene Hart Hospital, The Netherlands; Noortje Briët-Schipper, Groene Hart Hospital, The Netherlands; Yotam Raz, Groene Hart Hospital, The Netherlands; Ayesha Lavell, Hospital Amstelland, The Netherlands; Fatima El Morabit, Hospital Amstelland, The Netherlands; Gert-Jan Timmers, Hospital Amstelland, The Netherlands; Ad Dees, Ikazia Hospital, The Netherlands; Ginette Carels, Ikazia Hospital, The Netherlands; Berit Snijer, Jeroen Bosch Hospital, The Netherlands; Anne Floor Heitz, Leiden University Medical Center, The Netherlands; Pim A.J. Keurlings, Maas Hospital Pantein, The Netherlands; Susan Deenen, Maas Hospital Pantein, The Netherlands; Patricia M. Stassen, Maastricht University Medical Center, The Netherlands; Hajar Kabboue, Máxima MC, The Netherlands; Ineke Schouten, OLVG location East, The Netherlands; C.E.H. Siegert, OLVG location West, The Netherlands; Jacobien J. Hoogerwerf, Radboud University Medical Center, The Netherlands; Lianne de Kleijn, Radboud University Medical Center, The Netherlands; Frank H. Bosch, Rijnstate Hospital, The Netherlands; Annebel Govers, Sint Franciscus Gasthuis, The Netherlands; Bianca van den Corput, Sint Franciscus Gasthuis, The Netherlands; Susan Deenen, St. Anna Zorggroep, Geldrop/Eindhoven, The Netherlands; H.S. Noordzij-Nooteboom, The Van Weel-Bethesda Hospital ,The Netherlands; M.J. Dekkers, The Van Weel-Bethesda Hospital, The Netherlands; Annemarie van den Berg, University Medical Center Groningen, The Netherlands; Jan C. ter Maaten, University Medical Center Groningen, The Netherlands; Dennis G. Barten, VieCuri Medical Center, The Netherlands; VieCuri Medical Center, Tessel Zaalberg, The Netherlands; John Soong, National University Hospital, Singapore; Norshima Nashi, National University Hospital, Singapore; Louise S van Galen, Singapore General Hospital, Singapore; Lim Wan Tin, Singapore General Hospital, Singapore; Tharmmambal Balakrishnan, Singapore General Hospital, Singapore; Siti Khadijah Binte Zainuddin, Singapore General Hospital, Singapore; Christian H Nickel, University Hospital Basel, Switzerland; Victoria Siegrist, University Hospital Basel, Switzerland; Fraz Mir, Addenbrooke’s Hospital, Cambridge University Hospitals, United Kingdom; Channa Vasanth Nadarajah, Basingstoke and North Hampshire Hospital, Hampshire Hospitals NHS Trust, United Kingdom; Aled Lewis, Glan Clwyd Hospital - NHS Direct Wales, United Kingdom; David Ward, Hinchingbrooke Hospital, North West Anglia NHS Foundation Trust, United Kingdom; C Weerasekera, Hull Royal Infirmary, United Kingdom; Thandar Soe, James Paget University Hospitals NHS Foundation Trust, United Kingdom; Thomas Cozens, Royal Gwent Hospital, United Kingdom; Joanne McDonald, Salford Royal Hospital NHS FT, United Kingdom; Mark Holland, Salford Royal Hospital NHS FT, United Kingdom; Andrew Down, Southmead Hospital, North Bristol NHS Trust, United Kingdom; Immo Weichert, Ipswich Hospital, East Suffolk and North Essex NHS Foundation Trust (ESNEFT), United Kingdom; Harith Altemimi, The Queen Elizabeth Hospital, King's Lynn. NHS Foundation Trust, United Kingdom; Tim Cooksley, University Hospital of South Manchester, United Kingdom; A Seccombe, University Hospitals Birmingham, United Kingdom; Chris P Subbe, Ysbyty Gwynedd Hospital, United Kingdom; Ben Lovell, University College London Hospitals, United Kingdom; Colin Graham, Chinese University of Hong Kong, Hong Kong; Ronson Lo, Chinese University of Hong Kong, Hong Kong; Ling Leung, Chinese University of Hong Kong, Hong Kong; Rachel M Kidney, St. James’s Hospital, Ireland.
